# Report on the Sanitary Condition of Chicago

**Published:** 1858-07

**Authors:** N. S. Davis


### REPORT ON THE SANITARY CONDITION OF CHICAGO, FROM JAN. 1st TO JULY 1st, 1858; ALSO SOME COMMENTS ON THE NECESSITY OF A MORE EFFICIENT ORDINANCE IN RELATION TO CERTIFICATES SPECIFYING THE CAUSE OF DEATH, BEFORE BURIAL.

BY N. S. DAVIS.

(Read to the Cook County Medical Society, July 6th, 1858.)

The winter of 1857–58, was remarkable for the mildness of its temperature, the little fall of either snow or rain, and a general exemption from severe or fatal sickness. The spring months presented no striking peculiarities, until the middle of May, when an unusual fall of rain commenced, and continued until the second week in June. The amount of rain which fell throughout the Western and North-Western States was such, as to completely inundate large tracts of country, and cause the Mississippi river and its tributaries to rise higher than at any time previous during the last half century.

When the rains ceased for a brief period, the atmosphere was almost uniformly warm and relaxing, with a prevalence of south winds. About the middle of June the rainy season ceased, and was followed by two weeks of unusually high temperature, with winds almost constantly from the south or south-west. Atmospheric electricity was abundant, as indicated by frequent and vivid flashes of lightning; and the proper atmospheric moisture was not as great as in some other seasons, when there occurred a much less fall of rain. High winds prevailed several times during the last week in May, and on the evening of the 30th it amounted to a severe tornado, almost destroying a small town in the interior of the State. These facts are here noted for future reference, rather than for any connection they may have with the sanitary condition of this city.

The local influences which would affect the health of the city have not been materially different during the last six months from those of preceding years. The filth accumulated in the streets and alleys during the winter has not been as promptly removed, and the gutters opened, as is desirable. But the frequent and copious rains during May and the first ten days of June, kept the surface washed and the surface soil saturated with fresh water so constantly, that no immediate injurious effects could result. The general health of the city during the time embraced in this report, has been good; the average monthly mortality having been a fraction more than 132, or 793 for the six months. Compared with the five preceding years, the result is as follows:

| | 1853. | 1854. | 1855. | 1856. | 1857. | 1858. |
|---|---|---|---|---|---|---|
| January, | 38 | 112 | 123 | 110 | 138 | 117 |
| February, | 68 | 101 | 103 | 103 | 106 | 126 |
| March, | 81 | 95 | 123 | 92 | 124 | 156 |
| April, | 59 | 103 | 105 | 108 | 165 | 116 |
| May, | 73 | 147 | 90 | 134 | 117 | 139 |
| June, | 82 | 341 | 87 | 121 | 126 | 139 |
| | 401 | 899 | 631 | 668 | 776 | 793 |

The greater mortality in May and June, 1854, was occasioned by the epidemic cholera, which prevailed severely during that summer, and commenced as early as the last week in April.

Aside from this, the annual increase of mortality has been pretty uniform, and in strict conformity to the increase of population. So far the statistics of mortality are doubtless correct, and it would be of much interest and importance if we could state in addition the causes of death, in such a manner, as to show both the absolute and relative prevalence of all the more important forms of disease.

Unfortunately, however, the ordinance of the city, intended to compel the procurement of a certificate setting forth the cause or causes of death in all instances before burial, has been so imperfectly executed, as to be of no real value.

And the statements generally published in the daily papers each month, so far as they relate to the *causes* of death, are wholly unreliable. Thus, in the list of diseases reported for February last, we have 30 deaths attributed to "consumption,"

and one to "disease of the heart;" while in March there are no cases of consumption reported, but in their place we have "heart diseases, 43." But the most objectionable feature of these monthly reports is the employment of terms that are meaningless or entirely indefinite, such as "congestion," "teething," "dropsy," "tumor," etc., etc. To say that an individual died from *congestion*, is about as intelligible as to say that he died because he could not live any longer. Almost every month a considerable number of deaths are attributed to *teething*. Thus, in February, 8, and in June, 6, were placed under that head; and during July, August and September, the number each month is stated as high as 25 or 30. I had supposed that the growth of the teeth in infancy and childhood was a process as strictly natural as the growth of the hair or nails, and consequently that it was just as absurd to attribute death to the one as to the other. That children, during the ordinary period of dentition, are much more liable to attacks of cholera infantum, diarrhœa, dysentery, marasmus and hydrocephalus, than at any other period of life, is undoubtedly true. But to report such cases, when they terminate fatally, as deaths from *teething*, is not only absurd, but calculated to sustain, in the popular mind, the very erroneous notion that, when a child is teething, a moderate diarrhœa is harmless and should not be interrupted. That very notion causes hundreds of children to be neglected every summer until they are reduced to skeletons, and beyond any reasonable hope of recovery. It is unnecessary to state that *dropsy* is, at this time, well known to be a mere symptom of some prior affection involving one or more of the important organs of the body. Hence, we might as well place *pain*, dyspnœa, or cough, in the list of causes of death, as "dropsy." I presume no intelligent physician uses words so meaningless, in giving certificates of death. On the contrary, these terms appear in the monthly reports from the records of the city sexton by an evasion of the ordinance in relation to the procurement of certificates.

Instead of requiring those who apply for burial to procure in all cases a regular certificate from the attending physician, the undertakers frequently take the statement of the family or friends in relation to the matter, and place it on the city sexton's record without any application to the physician whatever. This evil should be remedied without delay. The rigid enforcement of an ordinance requiring the certificate of an intelligent physician stating plainly the cause of death, before permitting a burial, is very important in two respects: First, the facts to be gathered from such certificates in relation to the prevalence and fatality of different diseases, at different seasons of the year, in connection with varying meteorological phenomena, and varying habits of different classes of people, would greatly facilitate investigations concerning the causes of disease; second, it would constitute a strong barrier in the way of committing crimes, especially poisoning, and render detection much more certain when such crime had been committed.

It is very desirable that our city authorities should see that a judicious ordinance is enacted on the subject, and that it be rigidly enforced.

There have been no diseases sufficiently prevalent in the city during the past six months to require notice, except perhaps an increased prevalence of eruptive diseases, and hooping cough. From the early part of winter to the present time, measles, scarlet fever, small-pox, and hooping cough, have been moderately prevalent; but at no one time sufficiently so to merit the name of epidemic. In the great majority of cases these diseases have been mild, both in their course and the sequela that are so prone to follow them. During the months of February and March, the cases of measles were much prone to become complicated with lobular pneumonia; while, during the latter spring months, diarrhœa and dysentery were much more frequent concomitants both of this disease and scarlet fever. I find in the list of deaths for June *four* attributed to measles. The only fatal case which came under my observation was in February, and was the result of pneumonia, in a child six or eight months old; and it is fair to presume that all the deaths reported as resulting from measles, were owing directly to either pulmonary or intestinal complications. The deaths from scarlet fever during the last six months have averaged about 15 per month. The smallest number was in February, when only 4 were reported, and the largest in March, when the table presented 23. The general character of the disease was that of scarlatina simplex. Only a small proportion of the cases presented marked anginose symptoms, and a still smaller number those of a malignant character. Of the latter, four came under my own observation. They all occurred in one family, living in the northwestern part of the city. The parents were natives of Scotland, and occupied a small wooden house with a low chamber, in which they with their three children slept. The first child attacked was a girl five years of age. The commencement of the fever was sudden and severe, and was accompanied by general convulsions. An "Eclectic" physician was called, who treated the child for *worms*. On the morning of the fourth day I was called, and found the child dying.

Its neck and tonsils were swollen so as to obstruct the breathing; the tongue was dark-red and dry; lips a leaden paleness; eyes suffused; pulse threadlike and too frequent to be counted; the skin a livid or purplish color and covered with an efflorescence sufficiently characteristic to leave no doubt about the diagnosis. She ceased to live in about one hour after my visit. During the sickness of this child, a relative came and took lodgings in the same house with his wife and two children. About one week after the death of the first child, both the remaining children of the family, one a girl about seven, the other a boy two and a half years old, and the oldest child of the visiting family, a boy aged two and a half years, were all attacked nearly at the same time, and with great violence. I was immediately called to visit them, and found the two little boys vomiting frequently, with signs of great distress in the epigastric region, the glands of the neck moderately swollen; the eyes suffused; the pulse extremely rapid; skin dry and hot; breathing short and hurried, and frequent nervous twitchings. Before the end of the first twenty-four hours both these patients had repeated convulsions.

During the second day the glands of the neck became considerably more swollen; the pulse continued over 160 per minute, and in one it was full and firm, accompanied by a very red and hot skin; while in the other the pulse was smaller, skin less red and hot, but both were very somnolent or inclined to stupor. The girl, who was attacked at the same time, presented a very different set of phenomena.

The glands of the neck were slightly swollen; the face was pale; prolabia leaden color; extremities cool; and her mind and brain so free from disturbance that a bystander would suppose she was not seriously sick. She was, however, very restless, occasionally inclined to vomit; the heart's action frequent but feeble; the pulse scarcely perceptible at the wrist; and the whole capillary circulation very feeble.

During the latter part of the day the eruption began to make its appearance, and on the morning of the third day it was well developed over the whole surface in each case. There was no abatement of the symptoms, however, and the two boys died almost simultaneously on the morning of the fourth day, and the girl on the evening of the same.

The treatment pursued in these three cases was quite diverse. In the girl, the functions of the circulating system were so depressed from the beginning, that stimulants, quinine, and chlorate of potassa, were the principal remedies.

One of the boys presented so high a grade of feeble action, with a full pulse, and active cerebral symptoms, that with the advice of a medical friend I pursued an anti-phlogistic course, consisting of leeching the temples, cold applications to the head, alteratives and laxatives; while in the other boy the treatment was expectant; the most active remedy used being small doses of belladonna.

Yet the result was the same in all; and probably from the same cause, namely, the direct action of the fever poison upon the blood and the great centres of the ganglionic nervous system. Although small-pox and hooping cough have both been more prevalent than usual, especially during the months of May and June, yet the cases that have come under my care have presented no peculiarities worthy of record. As before stated, during the last half of June, the weather was continuously hot and oppressive with a prevalence of south and south-west winds.

During this time three deaths were reported from *sun-stroke*, and four or five cases of violent cholera morbus, accompanied by cramps in the muscles of the extremities, rice-water discharges, and many phenomena identical with epidemic cholera. But I am not aware that any of the cases proved fatal; and during the last few days, all intestinal disturbances have showed a tendency to dysentery. So far as an opinion can be formed from the characteristics of the season embraced in this report, I should predict a considerable prevalence of dysentery during the summer, and accompanied by remittent fever in the autumn.

### UNIVERSITY OF LOUISVILLE.

The faculty of the above institution has undergone a complete metamorphosis, and is constituted at present of the following able corps of teachers: Lunsford P. Yandell, M. D., professor of physiology and pathological anatomy; Benjamin R. Palmer, M. D., professor of the principles and practice of surgery; J. Lawrence Smith, M. D., professor of medical chemistry and toxicology; Robert J. Breckinridge, M.D., professor of materia medica and therapeutics; Joshua B. Flint, M. D., professor of clinical surgery; Theodore S. Bell, M. D., professor of the theory and practice of medicine; Llewellyn Powell, M.D., professor of obstetric medicine; J. W. Benson, M. D., professor of descriptive and surgical anatomy; S. M. Bemiss, M. D., professor of clinical medicine; Archie B. Cook, M.D., demonstrator of anatomy.

Dr. J. W. Benson, former professor of surgery in the Oglethorpe Medical College, Georgia, has resided here for the last three-quarters of a year. We wish him success in his new home and position. B.

### A MISTAKE.

The *New Orleans Medical News and Hospital Gazette* says: "The *Peninsular Journal* and the *Medical Independent* have been merged in one, under the title of the CHICAGO MEDICAL JOURNAL." The product of the above union is the *Peninsular and Independent Medical Journal*. It is published at Detroit, Mich., and could not with any propriety be called by our name.

B.

We would call attention to our advertising sheet as affording matter of much interest. If Prof. Eberle should wake up—although he has slept but for a few years, and was one of the most erudite physicians of his age—we doubt whether he would be able to write a prescription that would suit the present state of pharmacy. We were in at J. H. Reed & Co., 144 & 146 Lake street, the other day, when we witnessed with delight the results of pharmaceutical achievements, in the great variety of pharmaceutical granules and dragees (sugar-coated pills) of Garnier, Lamoureux & Co. of so delicate a character and delicious taste, as to put to shame all the sweet nonsense of homœopathy even for the palate.

These granules and dragees are medicines brought down in bulk to the most highly-concentrated forms, and enveloped in a coating of sugar. It will be seen, therefore, that they are entirely reliable, while they are divested of all offensiveness, two great desiderata.

The excellent syrups, concentrated fluid extracts, resinoids and alkaloids kept by Gale Brothers, 202 Randolph street, Sargent & Ilsley, 140 Lake street, are also preparations to which we would call attention as reliable and trustworthy. These established and faithful business houses may all be confided in by any persons interested in this line of business. In speaking of syrups, we do not mean to endorse any compound that is not officinal and well understood by the profession generally. We think no physician who holds his reputation as of much value, will long delay at least a trial of these (as we consider) improved forms of materia medica. As will be seen, they advertise a good assortment of instruments always on hand. Reed & Co. also advertise the prices for which they may be forwarded to physicians, by their merely sending the money with specific orders as to the kind desired. B.

### LONDON LANCET.

We see by advertisement on the cover of the *Lancet*, that twelve lectures delivered in London by the distinguished physiologist, Dr. Brown-Sequard, will be published in that periodical.

The subjects treated of are very interesting, and of course will be ably managed by the author. Now is the time to subscribe for the *Lancet*, that you may secure these excellent effusions of genius. The subscription price is only five dollars, and one number is worth the money very often.

New subscribers may begin with the beginning of the present volume. The first number is issued in July.

Address James Herald, proprietor of *London Lancet*, 14 Ann street, New York.

Stringer & Townsend having dissolved partnership, are no longer the publishers. B.

"To our exchanges we must say, those out west, as at Columbus, Ohio and St. Louis, which have a way of copying from the *Monthly* without credit, are requested to be more careful."—*American Medical Monthly.*

To our exchanges *we* must say, that those in Gotham, as the *American Medical Monthly*, which has a way of copying our translations without credit, should be more careful in future.

B.

PROFESSORSHIPS.

Prof. S. H. Dickson, of Charleston, S. C., has been called to the chair of theory and practice of medicine, made vacant by the death of the venerable J. K. Mitchel, M. D. Prof. Dickson will no doubt sustain the dignified position of that place.

P. C. Gaillard, M. D., of Charleston, was elected to fill the chair of institutes and practice in the Med. College of the State of South Carolina, made vacant by the resignation of Professor Dickson. J. J. Chisholm, M. D., of Charleston, was elected at the same time and place to fill the chair of surgery, resigned by Prof. Goddings of that institution. B.

Dr. B. L. Jones, of Georgia, is chosen to fill the chair of chemistry in Oglethorpe Medical College, and Dr. E. F. Colzey has taken the chair of physiology in that college. Both of these men are untried as professors, but represented as of superior qualifications, and will undoubtedly be a credit to their newly-assumed offices. B.

ILLINOIS STATE MEDICAL SOCIETY.

The treasurer of this society informed us a few days since, that the funds received for annual assessment, at the meeting recently held in Rockford, were barely sufficient to pay up the indebtedness of last year, leaving nothing to pay for publishing the Transactions of the present year.

Circulars have been sent to all the members informing them of the amount of assessment, and if they wish to have the Transactions published in proper time, they should send the amount ($3) to the treasurer, Dr. J. W. Freer, of Chicago, without delay.

BILLS.

With this number we send bills to part of our subscribers, and shall send to the rest next month. All the bills are made to include the present volume, ending with December, 1858. If any discover errors in their accounts as presented, we shall be most happy to correct them on being informed of the facts. Meantime, let all read the statement in relation to the *Journal* in another column and act accordingly.

CORRECTION.

The *Report on the Sanitary Condition of this City* was prepared for the meeting of the Medical Society, at which it purports to have been read. But professional engagements detaining the writer from the meeting, it was not presented. It being intended as the beginning of a monthly series for the remainder of the year, we publish it without waiting for another meeting of the Society.

